# A Microfluidic Device for Automated High Throughput Detection of Ice Nucleation of Snomax^®^

**DOI:** 10.3390/mi12030296

**Published:** 2021-03-11

**Authors:** Priyatanu Roy, Margaret L. House, Cari S. Dutcher

**Affiliations:** 1 Department of Mechanical Engineering, University of Minnesota—Twin Cities, Minneapolis, MN 55455, USA; royxx299@umn.edu; 2 Department of Chemical Engineering & Materials Science, University of Minnesota—Twin Cities, Minneapolis, MN 55455, USA; house331@umn.edu

**Keywords:** microfluidic device, ice nucleating particle, Snomax^®^, high-throughput, automated detection, machine learning, deep neural network, polarized light

## Abstract

Measurement of ice nucleation (IN) temperature of liquid solutions at sub-ambient temperatures has applications in atmospheric, water quality, food storage, protein crystallography and pharmaceutical sciences. Here we present details on the construction of a temperature-controlled microfluidic platform with multiple individually addressable temperature zones and on-chip temperature sensors for high-throughput IN studies in droplets. We developed, for the first time, automated droplet freezing detection methods in a microfluidic device, using a deep neural network (DNN) and a polarized optical method based on intensity thresholding to classify droplets without manual counting. This platform has potential applications in continuous monitoring of liquid samples consisting of aerosols to quantify their IN behavior, or in checking for contaminants in pure water. A case study of the two detection methods was performed using Snomax^®^ (Snomax International, Englewood, CO, USA), an ideal ice nucleating particle (INP). Effects of aging and heat treatment of Snomax^®^ were studied with Fourier transform infrared (FTIR) spectroscopy and a microfluidic platform to correlate secondary structure change of the IN protein in Snomax^®^ to IN temperature. It was found that aging at room temperature had a mild impact on the ice nucleation ability but heat treatment at 95 °C had a more pronounced effect by reducing the ice nucleation onset temperature by more than 7 °C and flattening the overall frozen fraction curve. Results also demonstrated that our setup can generate droplets at a rate of about 1500/min and requires minimal human intervention for DNN classification.

## 1. Introduction

The liquid-to-solid phase transition of a droplet has critical applications in pharmaceutical and food industries, where the formation of ice at low temperatures is an important determinant in the degradation of drug and food products during processing and storage [1,2,3]. Ice crystals also have a detrimental effect on cryopreserved biomaterials by causing mechanical damage and viability loss during storage or retrieval [4,5]. Nucleation and crystal growth at warmer temperatures also has implications in salt damage to building structures [6], protein purification [7] and crystallographic studies for high throughput screening [8].

But perhaps one of the biggest impacts of droplet phase change on human lives is caused by ice nucleation in aqueous aerosols in the atmosphere. In general, the phase state of aerosol particles can affect the climate directly by governing water uptake, scattering and absorption of solar radiation and indirectly by aerosol-cloud interactions [9,10,11,12,13,14]. Cloud microphysical processes and the cloud phase (liquid, mixed or ice) are governed to a large extent by nucleation of ice in cloud drops [14,15]. The phase of clouds, in turn, affects the water content and optical properties of the atmosphere [10,16].

Ice nucleation in cloud droplets usually occurs at temperatures well below 0 °C which is the thermodynamic freezing point of pure water. Clouds can be supercooled down to −38 °C before ice nucleates in water droplets homogeneously [17]. In addition, foreign solid particles or macromolecules internal to the droplets can act as heterogeneous nucleation sources or ice nucleating particles (INPs) by facilitating ice formation at temperatures anywhere in the range of −38 °C to 0 °C [18]. Commonly, INPs are submicron particles generated from terrestrial biological, urban, mineral dust or marine emission sources [18,19,20,21,22,23].

Ice nucleation temperature of INPs are measured using online or offline instruments. Continuous flow diffusion chambers [24,25], and continuous flow tubes [26] are typical online equipment which continuously measure INPs at a fixed temperature by connecting to air sampling lines from the ambient environment. However, they are complex and expensive. Among the offline equipment, large cloud expansion chambers can be used to simulate whole clouds in the upper troposphere [27]. However, there are other less complex and expensive classes of benchtop offline experiments which allow ice nucleation studies in a laboratory. For instance, differential scanning calorimeters [28], environmental Raman stages [29,30], electrodynamic balances [31], optical traps [32], static cold plate based freezing assays using well plates [33,34], printed droplet arrays on hydrophobic substrates [35], droplets in microwells [36] and droplet arrays on a pyroelectric polymer substrate [37] have been used to study ice nucleation from atmospheric and laboratory samples.

Recent advances in droplet microfluidic technology have allowed generation and study of phase changes of nano- to picoliter droplets for aerosol science applications [38,39,40,41,42,43,44,45]. These devices range from microfluidic droplet generators for off-chip analysis [42,46], and on-chip analysis with static arrays of droplet traps [45,47] to flow-through channel based devices [43,48]. Flow-through stages can be particularly advantageous by generating droplets continuously throughout an experiment, allowing for high-throughput studies on a large number of droplets, and removing any potential contact with a solid surface or adjacent droplets by completely enveloping the target droplet in a liquid carrier phase. Additionally, these stages can be custom designed for sorting frozen droplets from liquid [44], enabling recovery of the droplets for downstream analysis. A disadvantage is that these droplets experience much higher droplet cooling rates when compared to typical rates experienced by cloud drops in the atmosphere. In all these devices, droplet freezing detection can be performed using bright-field microscopy, either through a brightness threshold or manual inspection and counting. For a review of microfluidic phase detection devices including INP counters, see Roy et al. [49].

Here, we document the initial steps towards the development of a microfluidic platform with on-chip droplet generation and continuous high-throughput ice nucleation detection. The platform and methods described here could be adapted to count ice nucleating particles in atmospheric aerosol samples collected in liquid. Here the high throughput nature of the device would allow for measuring minuscule amounts of INP in field samples more accurately at warmer temperatures. The stage could also be modified into a new class of liquid particle counter, analogous to condensation particle counters for aerosols, where a small number of particles in ultrapure liquid phase samples can be detected by measuring ice nucleation temperatures of a large number of droplets rapidly. One should note, however, that while there are advantages to high-throughput methods, there are also limitations, including difficulty in accessing low cooling rates (~1 °C/min) as well as observing phase change events in individual droplets with highly precise temperature measurements.

We present two automated detection approaches: a polarized light method and a deep neural network (DNN) based machine learning method. The appropriate detection algorithm is implemented depending on the throughput rate needed, and optical contrast available. We use this stage and our algorithms to demonstrate the methods’ feasibility in a case study using Snomax^®^ [50] which is a well characterized commercial ice nucleating particle, and acts as a “warm” INP able to nucleate ice efficiently above −10 °C. We also use spectroscopic techniques to investigate the effects of aging up to 45 days and thermal treatment up to 95 °C on the secondary structure of the ice nucleating protein in Snomax^®^, following protocols developed in previous studies on biomaterials [51,52,53]. We correlate the ice nucleation temperature with the treatments. To our knowledge, this is the first high-throughput microfluidic device of its kind where automated machine learning and polarized light-based detection algorithms have been used to detect ice nucleation.

## 2. Materials and Methods

### 2.1. Temperature-Controlled Platform

**Figure 1:**
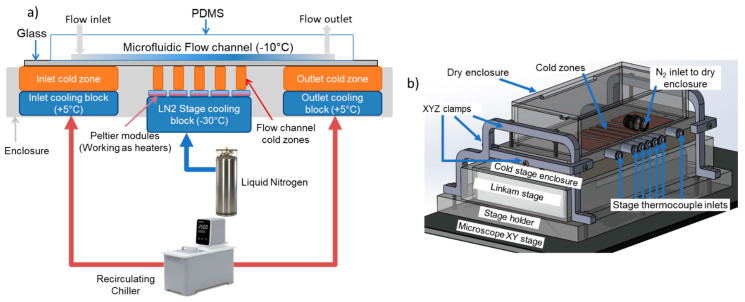
Temperature-controlled microfluidic platform: (**a**) Schematic showing the complete platform with multiple copper blocks for individually controllable cold zones and the cooling blocks underneath which act as heat sinks for the cold zones; (**b**) CAD model showing the platform on a microscope stage with a dry, optically transparent enclosure on top surrounding the region where the microfluidic device is placed.

A temperature-controlled platform based on Stan et al. [48,54] was constructed with multiple independent temperature zones, to control the droplet temperatures inside the microfluidic channel. The design allows a temperature gradient along the flow channel. Figure 1a shows our platform design with seven upper copper blocks (called cold zones) with the microfluidic device placed on top. The cold zones are in thermal contact with separate heat exchangers (called cooling blocks) with cooled liquid flow going through them to maintain a fixed temperature.

Two separate sets of cooling blocks are used. The inlet and outlet cold zones are in contact with two cooling blocks maintained at above freezing temperatures by a recirculating chiller (Anova R10, Anova Industries, Houston, TX, USA), pumping ethanol at 5 °C through the cooling blocks. The flow channel cold zones are in contact with a programmable cooling block (LTS 420, Linkam Scientific, Tadworth, UK) maintained at cold temperatures (−30 °C) by pumping liquid nitrogen through the block. Thermal grease (Ceramique 2 Thermal Compound, Arctic Silver, Visalia, CA, USA) was applied between all mating surfaces to ensure adequate heat transfer at low temperatures.

**Figure 2:**
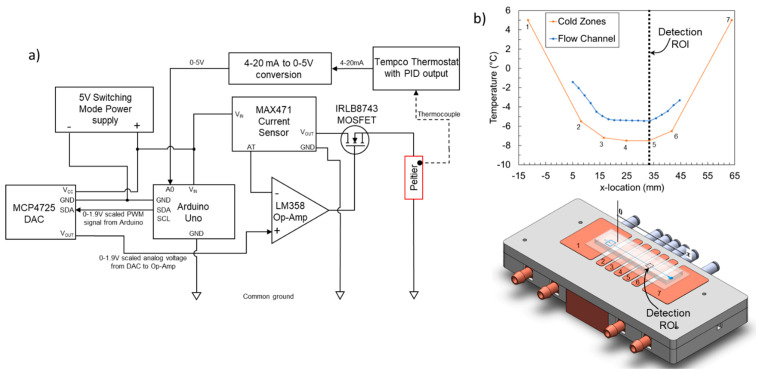
(**a**) Custom designed control circuit for the temperature-controlled platform (**b**) Cold zone temperatures measured using thermocouples and microfluidic channel temperatures measured using on-chip thin film sensors. The number labels next to the orange line indicate the corresponding cold zone in the schematic, and the temperature values are measured using thermocouples inserted into the copper blocks. The blue line indicates the temperature in the flow channel. Also shown is the location of the detection region of interest (ROI) during droplet freezing experiments.

Sandwiched between the flow channel cold zones and the liquid nitrogen cold block are Peltier elements (926-1209-ND, Laird Technologies, Durham, NC, USA). These elements act as temperature controllers and maintain the desired temperature in the cold zones on top, independent of the liquid nitrogen cold block temperature underneath. A custom PID loop based control circuit was built using both discrete electronic components and off-the-shelf PCBs. T-type thermocouples (TJC36-CASS-062U-2, Omega, Norwalk, CT, USA), were inserted into holes drilled into each cold zone. Thermocouple readings were taken with thermostats (TEC-9100, Tempco, Wood Dale, IL, USA). Target values for each cold block were selected and the thermostat provided a PID control signal to the Peltier elements to maintain the target temperature. This thermostat output was insufficient to power the Peltier elements and hence this signal was amplified using the circuit shown in Figure 2a. Briefly, the 4–20 mA current signal was first converted to a 0–5 V voltage signal. This voltage signal could not be directly converted to a 0–5 A current and fed to the Peltier elements since they operate within a 0–1.9 A current limit. An Arduino Uno (Ardunio.cc) was used to scale the 0–5 V signal to a 0–1.9 V PWM signal which was converted to a smooth DC signal using a digital-to-analog converter, or DAC (MCP4725, Adafruit, New York, NY, USA). A 0–30 V lab benchtop DC power supply (Amazon.com, Seattle, WA, USA) was then used to drive the Peltier elements by modulating its output to a 4.5 V, 0–1.9 A current source using a current sensor (MAX471, Maxim Integrated, San Jose, CA, USA), Op-Amp (LM358, Texas Instruments, Dallas, TC, USA) and an n-channel MOSFET (IRLB8743, Infieon, San Jose, CA, USA).

Figure 2b shows the temperatures in all the cold zones during a typical experiment. The temperature of each cold zone is adjusted until a desired temperature distribution is achieved inside the microfluidic channel (See Section 2.2 for details on microfluidic channel temperature measurement). Droplet freezing detection was performed using this stage on a reflective microscope (SZX10, Olympus Life Science, Waltham, MA, USA) and 128 × 256 pixel videos were recorded with a high-speed camera (AX100 mini, Photron, San Diego, CA, USA) at 3000 frames per second. A region of interest (ROI) of 250 μm × 500 μm was recorded for a continuous 4 min of experiment time, and a constant temperature was maintained in the flow channel.

### 2.2. Platinum Thin Film Temperature Sensor Design and Fabrication

**Figure 3:**
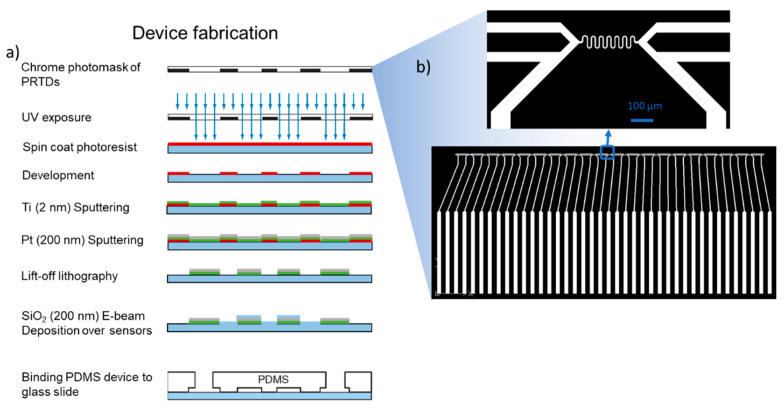
PRTD array fabrication and integration with microfluidic poly-dimethylsiloxane (PDMS) channels. The design is based on Stan et al. [48]. (**a**) PRTD array fabrication and bonding process schematic; (**b**) Array mask design, showing 19 PRTD arrays in a row.

Embedded thin film temperature sensors similar to those in Stan et al. [48] were used. Figure 3a,b shows the design and fabrication process of the platinum resistive temperature device (PRTD) linear array. These arrays were fabricated onto 4” diameter and 0.5 mm thick soda-lime glass wafers (University Wafer, Boston, MA, USA) using a modified version of the lift-off lithography process described in [48,55]. Briefly, the wafer was spin coated with a positive photoresist (AZ 1518, Microchemicals GmBH, Ulm, Germany), a chrome photomask of the PRTDs was used (UMN MNC) to expose the coated wafer to UV light, and the exposed PRTD regions were developed away. Then a 2 nm adhesion promoter layer of Ti, followed by a 200 nm layer of Pt were sputter coated onto the wafer. The array had 19 PRTDs in a row and a pitch of 2.2 mm between each PRTD. The wafer was annealed for 24 h at 500 °C in a furnace to stabilize the metal layer resistivity and extend its lifespan.

To measure the resistance of the PRTD array, a custom designed printed circuit board with 40 contact pads (Sunstone Circuits, Mulino, OR, USA) was attached to the leads on the substrate using electrically conductive adhesive transfer tape (9703, 3M, Saint Paul, MN, USA) which only conducts electricity across its thickness through embedded discrete conductive particles. A programmable digital multimeter (Keithley 2701 with a 7710 multiplexer card, Tektronix, Beaverton, OR, USA) was used to read individual PRTD voltages which were converted to resistance using a modified four-wire protocol [48] implemented in LabVIEW. The fabricated sensors were calibrated against a factory calibrated standard PRTD (5606-50-B, Fluke Calibration, Everett, WA, USA). Further calibration details are given in the ESI. The accuracy of the sensor array from the calibration curve was ±0.03 °C.

### 2.3. Microfluidic Device Design and Fabrication

**Figure 4:**
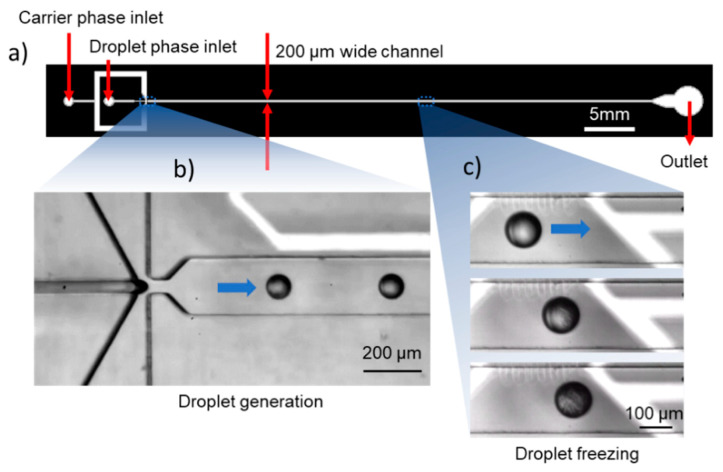
(**a**) Microfluidic channel design for the study; (**b**) Droplet generation at the flow-focusing junction; (**c**) Droplet ice nucleation and complete crystallization observed in the cold zone. The white region in the background is the temperature sensor underneath the flow channel.

The microfluidic device we used is a simple straight channel with a flow-focusing droplet generator [48]. The main channel dimensions are 200 × 150 µm and has a length of 50 mm. The width of the droplet phase channel upstream of the flow-focusing junction is 40 µm. The device design is shown in Figure 4a. A negative vinyl mask was printed (CAD art services), and the mold for the polydimethylsiloxane (PDMS) devices was prepared in the UMN MNC cleanroom using the following modified photolithography process: Solid state SUEX sheets (DJ Microlaminates, Sudbury, MA, USA) were bonded to standard 4” silicon wafers at 70 °C in a thermal laminator. The sheets act as negative photoresists, and standard photolithographic processes previously described [41] were used following this modification to develop the channel pattern on the wafer. The sheets were used to ensure height uniformity as spin coating was seen to cause slight alterations in the height across the long flow channel.

The glass substrate with the PRTD array was cut into 70 × 40 mm rectangles containing the sensors and the leads. The lead region was masked off with Kapton tape (3M) and a 5 µm layer of SiO_2_ was deposited onto the wavy sensor region using an e-beam deposition system. The PDMS device was cut out from the mold using a razor, and 1.5 mm diameter holes were punched for inlets and outlet with a biopsy punch (Integra Miltex Instruments, York, PA, USA) before being bonded to the substrate on the silica layer on top of the PRTDs following a 1-min oxygen etching in a plasma cleaner.

The device was bonded to the substrate with the PRTDs at an offset from the centerline of the flow channel ensuring the wavy sensing region of the PRTD did not sit directly beneath the flowing droplets as shown in Figure 4c. This bonding protocol prevents lensing of the sensor outline through the droplet from causing freezing detection errors in our DNN algorithm. Figure 4c also shows progression of the freezing front in a droplet, from the initiation of the droplet to complete crystallization. Events like this were rare, however, since the ROI in our studies was chosen so that droplets would freeze before entering the field of view of the camera.

Droplet generation was performed by feeding the sample of interest at the droplet phase inlet and a bulk oil phase at the carrier phase inlet (Figure 4b). The droplets were generated by the bulk phase pinching off the droplet phase at the droplet generation junction. Droplet size ranged from 70 to 85 µm in diameter and velocity ranged from 11.1 to 13.2 mm/s depending on the temperature but was constant at a given temperature. Additionally, the droplet spent ~1 s at the isothermal region inside the cold channel before reaching the detection ROI. This relatively long residence time spent inside this isothermal region ensures that the droplet reaches thermal equilibrium with the channel before entering the ROI. We verified this by numerically evaluating the temperatures inside the flow channel and the droplet with a two-step model using COMSOL Multiphysics (Comsol Inc., Burlington, MA, USA). The model shows that the droplet indeed equilibrates to the channel temperature inside the cold zone for all the temperatures the experiments were performed at. The simulations aid in confirming the accuracy of the measured temperature, though empirical measurements of melting points of pure liquids such as water and hydrocarbons would also aid in calibrating the droplet temperatures accurately. However, these were not attempted due to significant changes required in the stage and flow device to freeze and melt droplets inside the same channel. More details on the model and the temperature distributions can be found in the electronic Supplementary Information (ESI). Example videos showing droplet generation and freezing have been provided in the ESI.

### 2.4. Chemicals Used

Light mineral oil (CAS 8042-47-5, Sigma Aldrich, Munich, Germany) was used as the continuous phase in all the experiments and HPLC grade water (CAS 7732-18-5, Thermo Fisher Scientific, Waltham, MA, USA) was used as the dispersed phase. Snomax^®^ dissolved in the water was used as a model INP for our case study. Snomax^®^ was received in dry pellet form and stored in a −20 °C freezer between uses. On the day of the experiment, pellets were dissolved in water at a concentration of 1 mg/mL in 50 mL sterile centrifuge tubes (Nunc 50 mL, Thermo Fisher Scientific) by initially vortexing and then shaking manually until the solution became visibly homogeneous.

The Snomax^®^ solution and mineral oil were loaded into 10 mL gas tight glass syringes (SGE Inc., Melbourne, VIC, Australia). Between each run, the syringes were emptied and cleaned with methanol, distilled water and isopropanol and dried with N_2_ gas. Syringe filters (Millex 0.2 µm, Millipore Sigma, Munich, Germany) were added to filter out any large undissolved Snomax^®^ particles and contaminants such as fibers in the syringes. Polyethylene tubing with ID 0.047”, OD 0.067” (BD Intramedic, BD Medical, Franklin Lakes, NJ, USA) was used to connect the filter outlets to the microfluidic device. Syringe pumps (Harvard Apparatus, Cambridge, MA, USA) were used to pump the liquids inside the microfluidic device at a steady rate.

### 2.5. Aging, Heat Treatment and Fourier Transform Infrared Spectroscopy (FTIR) Study Methodology

Snomax^®^ samples were aged by dissolving the Snomax^®^ in solution and leaving the samples at room temperature for either 7 or 45 days before testing them in the microfluidic setup. Heat treatment was performed by preparing 10 mg/mL solutions of Snomax^®^ in cryovials (Corning, Tewksbury, MA, USA) and leaving the vials in a water bath at 55 °C or 95 °C for 20 min. Subsequently, the samples were diluted to a 1 mg/mL solution.

Finally, for the FTIR study, samples were prepared by dissolving Snomax^®^ in D_2_O (Sigma-Aldrich) at 1 g/mL which resulted in a highly viscous paste-like consistency. This process was developed to eliminate the H_2_O scissoring band overlap with the protein amide-I band as described in Liu et al. [52]. The samples were measured in an attenuated total reflectance (ATR) accessory (MVP-Pro, Harrick Scientific, Pleasantville, NY, USA). The ATR is equipped with a diamond sampling crystal and is internally purged with dry air. A mercury cadmium telluride detector cooled by liquid nitrogen was used for the study. Each spectrum was averaged over 64 consecutive readings measured with resolution 4 cm^−1^ in the wavenumber range 4000–650 cm^−1^. Omnic 9.2 software, supplied with the instrument, was used to fit the amide-I region (1600–1700 cm^−1^) of the spectra [51] with Gaussian peaks to isolate relative abundance of secondary structures of the *inaZ* protein in the samples following the treatments.

## 3. Results

### 3.1. Polarized Intensity Threshold Algorithm for Freezing Detection

The high-speed camera captured approximately 4000–6000 droplets in a single video of 4-min duration. To determine the fraction of frozen droplets at a given temperature and determine the ice nucleation rate, a method for counting the number of liquid and frozen droplets is needed. Ideally, the method is robust and high-speed, ultimately allowing for on-chip detection with the potential for downstream actuation and sorting. Here, we explored two methods; automated detection with polarized light images and with a deep neural network from bright field images. Both methods are benchmarked to simple manual counting from brightfield optical images.

**Figure 5:**
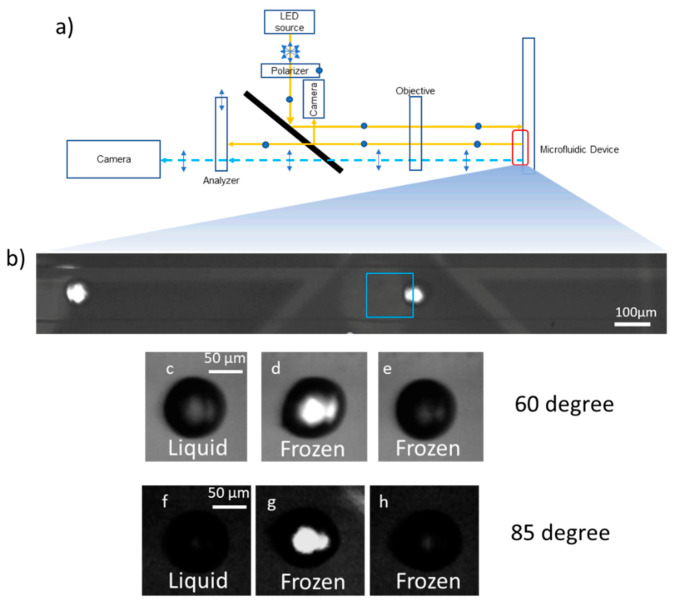
Experimental setup and freezing detection algorithm using a polarized intensity threshold method: (**a**) Polarized imaging optical path; (**b**) Region of interest highlighted in blue inside the flow channel; (**c**–**h**) Liquid, frozen (bright) and frozen (dark) droplets as they appear inside the region of interest for different analyzer angles indicated.

In the first method, a polarized detection scheme was used to detect droplets with ice crystals inside. Similar setups have been used previously to detect ice nucleation [56,57] in flow tubes and crystallization in polymer laden droplets [58]. The polarizer-analyzer set with rotatable filters (Edmund Optics, Barrington, NJ, USA) was installed in the optical path between the light source and the camera as shown in Figure 5a. The light source is polarized through a plane polarizer before passing through the droplets once. The light is then reflected from the mirror coating underneath the substrate before passing through the droplets a second time. Finally, it passes through the analyzer before reaching the camera. With perpendicular alignment of the analyzer with respect to the polarizer, almost all light passing through the liquid and PDMS layers is extinguished leaving a dark image. In this arrangement, only the frozen droplets show up as bright regions in the image due to the birefringence of the ice crystals. The birefringence changes the polarization angle of the light, which allows light to pass through the analyzer (Figure 5b). While use of 90° polarization is a straightforward method for rapid detection of droplets containing ice crystals, it poses problems in detecting liquid drops. Purely liquid droplets remain completely dark and blend in with the background, making it a challenge to detect the total number of droplets that pass through the channel. Additionally, some frozen droplets appear darker than liquid drops even in polarized light since the crystal plane orientation inside a droplet is random with respect to the polarization angle of the incident light (Figure 5e,h). To solve this issue, the analyzer was placed at 60° and 85°, to find the optimum balance between the intensity of the frozen drops and the ability to still identify liquid drops.

To partially automate the detection of the frozen droplets with this setup, a MATLAB image analysis code was created to detect and count frozen and liquid droplets under polarized light conditions. Briefly, the code is used to calculate two intensity threshold parameters based on either type of frozen drops, brighter or darker than liquid drops. The higher threshold was selected to identify droplets that are brighter than a liquid drop and vice versa for the lower threshold. These parameters are used to analyze the remaining video frames and give a final count of liquid and frozen droplets in a video, which are typically on the order of 4 h when played back at 60 fps. This code, which takes minutes to run, saves the user hours of analysis time that would have to be spent manually counting droplets. The polarized light detection code and usage instructions are given in the ESI.

### 3.2. Deep Neural Network Algorithm for Freezing Detection

In the second detection method, a DNN based on transfer learning using the well-known AlexNet [59] was chosen. This network, available in the MATLAB Deep Learning Toolbox, has 23 layers, 1000 object classes and has been pretrained with 1.3 million high-resolution images in the LSVRC-2010 ImageNet training set. We modified this network by keeping the convolution layers intact and replacing the final three layers with (1) a new fully connected layer, (2) a softmax layer, and (3) a classification layer with two classes: frozen and liquid. The modified network is called dropletnet and is trained with liquid and frozen drop images extracted from videos taken at the highest and lowest temperatures during the experiment. The training set temperatures were carefully chosen far away from the temperature range where the frozen fraction was expected to be between 0 and 1. Training was performed separately for each sample tested as we found this gave more accurate results. This was probably due to changes in the background light intensity and tiny shifts in position of the camera with respect to the channel between experiments. A custom MATLAB app was developed based on the algorithm.

**Figure 6:**
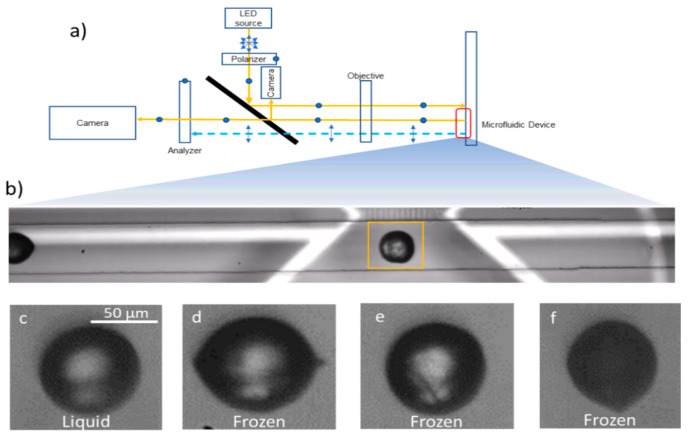
Experimental setup and freezing detection algorithm using a deep neural network: (**a**) Bright-field imaging optical path; (**b**) Region of interest highlighted in yellow inside the flow channel; (**c**–**f**) Liquid and different frozen droplets as they appear in bright field images.

The optical path for bright field imaging used the same setup as the polarized imaging, with the analyzer turned to 0 degrees with respect to the polarizer (Figure 6a). The different liquid and frozen droplet morphologies are shown in Figure 6c–f. It is clear from these images that a simple intensity threshold based freezing detection algorithm would do rather poorly in classifying liquid and frozen droplets and multiple parameters such as shape and intensity would be required. However, these parameters would also fail to distinguish between the clearly frozen droplet in Figure 6e from the liquid droplet in Figure 6c. As a result, we found that the DNN approach described here worked better than approaches where distinguishing features were manually extracted from the images. Details about the algorithm and usage instructions are given in the ESI.

**Figure 7:**
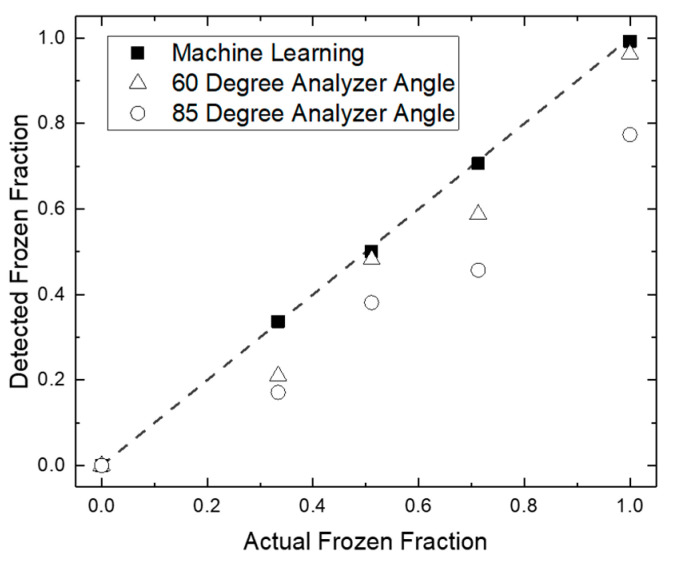
Comparison of the different methods for detecting frozen droplets. The dotted line denotes an ideal detection method i.e., where the method performs identical to a human operator.

The polarized method and the DNN methods for automated detection are compared to results obtained from simple manual counting of frozen droplets from optical images in Figure 7 at four different temperatures with Snomax^®^ samples. The machine learning DNN method yields results within 99.1 ± 0.7% accuracy compared to the manual method. In contrast, it is apparent from initial trials on Snomax^®^ samples at a few distinct temperatures that the polarized method is the less accurate of the two. The polarized light code underestimates the frozen droplet count at both 60° and 85° analyzer angles compared to manual and DNN count, discussed in Section 4. As a result, the DNN was used for all Snomax^®^ results presented in the following Section 3.3. The data in Figure 7 was recorded from an untreated Snomax^®^ sample and is separate from data reported in subsequent figures. While the polarized method proved less accurate DNN here, a discussion in Section 4 is provided about its potential for replacing the DNN in certain scenarios.

### 3.3. Case Study: Snomax^®^ Ice Nucleation Detection

**Figure 8:**
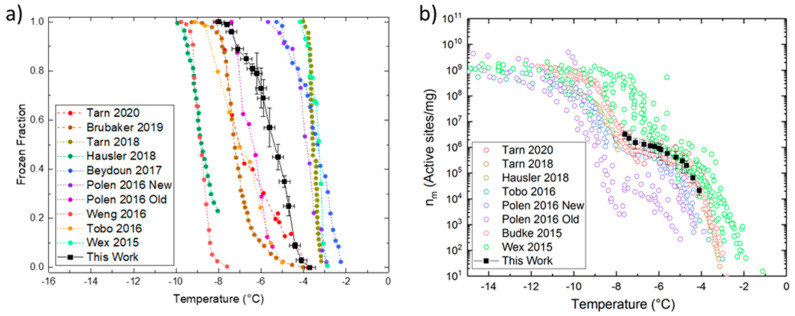
(**a**) Plot showing the frozen fraction of Snomax^®^ in our microfluidic setup vs. experimental data from literature [42,43,45,46,47,50,60,61,62]. The horizontal error bars in the data represent the combined temperature uncertainty of the droplets due to the temperature variation along and across the isothermal channel during experiments. The vertical error bars represent the standard deviation from three independent samples; (**b**) Plots showing the ice nucleation site density per unit mass of Snomax^®^ and comparison with literature data [36,42,43,47,50,60,61].

Figure 8 shows curves of frozen fraction from Snomax^®^ samples from our microfluidic set-up compared to other methods. The microfluidic experiment is performed by first setting the cold zones to a desired temperature and reading the resulting temperature inside the microfluidic channel. Adjustments to compensate for the thermal connection are made by either changing the individual set point temperatures for the cold zones and/or the temperature of the underlying liquid nitrogen cooling block until a uniform temperature is reached over most of the microfluidic channel. Once the thermal test conditions are reached, a video of the droplets is recorded, and the frozen fraction is calculated by the DNN using the formula:

(1) $$f\left(T\right)=\frac{N_{f}}{N}$$

where f(T) is the frozen fraction at temperature T, $N_{f}$ is the number of frozen droplets, and N is the total number of droplets.

This process was repeated over multiple temperatures to generate the curve shown in Figure 8a. Each point in the curve has data from three independent samples or around 18,000 droplets. The horizontal error bars indicate uncertainty in freezing temperature measurement and the vertical error bars indicate standard deviation of the frozen fraction measured. Compared to [43,47,60], our sample starts to nucleate ice at lower temperatures, at around −4 °C and does not reach a frozen fraction of 1 until −8 °C. However, this curve is in an overall warmer zone compared to [42,50,61]. The differences in the temperature ranges are due to the differences in the quality (or age) of the Snomax^®^ samples. Specifically, it was found that different batches of Snomax^®^ create significant variation in the ice nucleation onset temperature [50]. The measurement method used [61] can also introduce variability in the IN temperature measured.

Heterogeneous ice nucleation, such as the nucleation resulting from using Snomax^®^ as an INP here, can be modeled using both a deterministic time-independent approach or a classical nucleation theory based stochastic time-dependent approach [18]. Following methods employed by [42,43,50,61] for Snomax^®^ and for simplicity, we adopt the deterministic approach and report temperature dependent ice nucleation active site density $n_{m}\left(T\right)$ using the following equation [63]:

(2) $$n_{m}\left(T\right)=-\frac{\ln\left(1-f\left(T\right)\right)}{V\times C_{m}}$$

where, V is the droplet volume and $C_{m}$ is the Snomax^®^ concentration in the droplet. A comparative plot is shown in Figure 8b. It shows that our device captures the trend in Snomax^®^ samples quite well when compared to literature data recorded on different instrumentation. Snomax^®^ has been shown to be an aggregate of three different classes (A, B, C) of INPs which are responsible for nucleating ice at different temperature ranges [64]. Our Snomax^®^ sample freezes between −4.1° to −7.9 °C which indicates the presence of class A (freezes above −4.5 °C) and class B (freezes between −6.5 °C and −4.5 °C) INPs with some class C (freezes below −7.5 °C) INPs. Recent studies [61,65] postulated that class A and class C INPs dominate the population and there are very few class B INPs present in Snomax^®^, which explains the steep rise at the warmest and coldest regions of the curve with the intermediate plateau.

**Figure 9:**
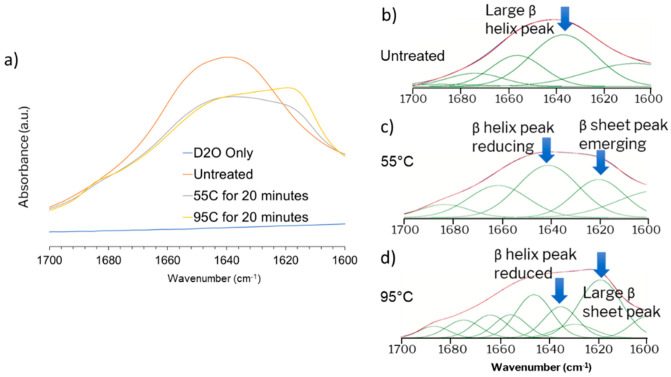
(**a**) FTIR spectra of D_2_O, untreated, 55 °C and 95 °C treated Snomax^®^ samples in the Amide-I region; (**b**–**d**) Peak resolve analysis of the amide-I region for distinguishing secondary structure of the ice nucleating protein *inaZ* in Snomax^®^ with the heat treatment conditions for the samples indicated in the image.

A previous study on Snomax^®^ by Polen et al. [50] studied the detrimental effects of long term storage, which also partially explains some of the variability in the freezing temperatures between results seen in Figure 8. Another study investigated antifreeze protein induced inhibition [65] of IN in Snomax^®^. We chose to study the effect of heat and aging at room temperature by heat treating Snomax^®^ samples at two different temperatures as shown in Figure 9a. FTIR spectra of the treated samples (Figure 9b) show that there is a significant difference between the positive control of untreated Snomax^®^, the negative control of D_2_O and the heat treated samples. This difference is significant in the Amide—I region which reveals the secondary structures of proteins (Figure 9c). A Gaussian peak fitting method was used to resolve the peaks in this region and reveals that the β-helix structure significantly decreases in the heat-treated samples and correlates with the heat treatment temperature with a concomitant increase in the β-sheet structure, indicating denaturation of the proteins.

**Figure 10:**
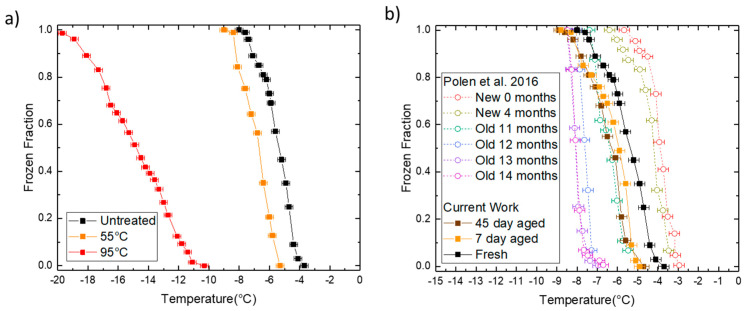
(**a**) A plot of the frozen fraction of heat treated Snomax^®^ samples as a function of freezing temperature; (**b**) A plot of the frozen fraction of room temperature aged Snomax^®^ samples as a function of freezing temperature.

Figure 10a shows the frozen fraction of heat-treated Snomax^®^ samples as a function of temperature in H_2_O measured with our microfluidic platform. The frozen fraction vs. temperature curve of the 55 °C treated sample moves about 1.3 °C towards the colder temperatures indicating partial deactivation of the active ice nucleating protein *inaZ* in Snomax^®^ due to initiation of heat-induced damage. The 95 °C treated sample shifts dramatically to the colder region, with a >7 °C decrease in onset and a flattened frozen fraction curve, indicating a drastic decrease in the ice nucleating ability of Snomax^®^, likely due to intensive thermal degradation of the ice nucleation sites.

Results of Snomax^®^ aging at room temperature are shown in Figure 10b. The ice nucleation temperature range from our study is comparable to Polen et al. [50]. Our samples were obtained more than a year before the experiments and stored in a freezer at −20 °C. Storage at such low temperatures appears to have reduced the negative impact of aging, compared to the results of Polen et al. at 12 months of age. Also, once taken out of storage, subsequent aging had some impact on the IN profiles, though there was no significant difference between the aged samples themselves. Our untreated or “freshly thawed samples” freeze at temperatures colder than the “new” samples from Polen et al. The 7-day and 45-day aged samples show about 1 °C lower freezing onset temperatures when compared to our untreated sample. Each aged and untreated data point on the curve in Figure 10 represents the mean of three repeated measurements on the same experimental sample. The error bars on the untreated sample have been omitted for consistency.

## 4. Discussion

As mentioned in the Results section, the polarized detection method undercounts the frozen fraction compared to both human and DNN count while postprocessing a video. This error occurs when the birefringence inside the frozen droplet is not pronounced. It is especially apparent in recalescent droplets [66,67,68], where the droplet becomes an ice-water slush due to the presence of both multiple kinetic ice crystals and supercooled water immediately after ice nucleation. These drops do not change the polarization angle of light appreciably due to their inhomogeneity and hence are almost indistinguishable from liquid drops in the polarized lighting setup. When observed under bright-field lighting, it is immediately apparent that these drops have nucleated ice. Additionally, due to the random nature of the ice crystal orientation inside the droplet and the bulk rotations in the droplets as a whole as they flow through the channel, it is possible that even some completely crystallized droplets do not show up as bright spots in the polarized light. This kind of crystal orientation rearrangement could be caused by either progressive crystal formation inside the already ice nucleated droplets or droplet bulk rotation inside the channel. Videos in ESI show this phenomenon both in bright field and polarized light.

Because of these shortcomings, the polarized method was not used to generate accurate datasets in our present device and working fluid combination. But we see other uses for this method, such as in a real-time active sorting device design where the frozen droplet must be detected at run-time for downstream actuation or sorting to take place. The droplet birefringence might be more pronounced in other combinations of carrier and droplet phases [43,48] which would aid the polarized method in becoming more accurate. This kind of approach would be economical, eschewing the need for a high-speed camera by using simpler 60 fps area scan cameras, or even using line scan cameras where a single line of pixels is captured at high framerates.

A potential disadvantage of flow-through microfluidic devices, when compared to static devices, is the high cooling rates experienced by the droplets as they cool while flowing through a fixed temperature gradient at high velocities. For example, in our setup the droplet spends around 3.3 s in total inside the device, out of which 1 s is spent inside the isothermal freezing region. This imparts a cooling rate on the droplet which is dependent on the cold region temperature, ranging from 140 °C/min at 0 °C to 720 °C/min at −20 °C. A previous flow-through microfluidic INP study by Tarn et al. [43] with similar droplet sizes, velocities and channel dimensions demonstrated that droplets with relatively short residence times (~0.2 s) inside a cold isothermal channel could still freeze in similar temperature ranges reported by instruments with orders of magnitude higher residence times. While ice nucleation by atmospheric particles is a stochastic process, it has been reported that highly efficient INPs including Snomax^®^ [36] can be approximated with the deterministic, time-independent approach we used for this study. Nevertheless, in order to adapt our device for studying atmospherically relevant particles, it would be valuable to study the effect of different cooling rates and residence times by modifying the cold stage and flow device to impart highly variable cooling rates.

We did not observe homogeneous freezing of ultrapure water down to −20 °C. Attempts to reduce the temperature further were unsuccessful due to the carrier fluid viscosity; the syringe pumps stalled trying to push the liquid through the microfluidic device. Notably, studies on flow-through microfluidic INP counters [43,48] with similar droplet sizes, velocities and microfluidic channel dimensions have measured homogeneous freezing of water between −35 °C and −37 °C. A different carrier fluid and redesigned channels with lower pressure drop could enable homogeneous freezing measurements in future INP studies. In the absence of the background water freezing curve, this present work is intended to highlight our platform, freezing detection algorithms and the relative effects of modifying the IN behavior due to aging and heat deactivation compared to untreated Snomax^®^.

Regarding the results using Snomax^®^, our case study shows that our current setup is capable of detecting ice nucleation with a very high throughput without manual intervention. The frozen fraction curves for the untreated and aged Snomax^®^ are comparable to literature data compiled using a multitude of other methods by Wex et al. [61] such as an acoustic levitator, a wind tunnel and droplet arrays on cold plate methods. Microfluidic approaches, both with surfactants to prevent droplet coalescence and stabilize droplets [42,43], and without surfactants [45], have been compared as well and show similar trends in terms of both the frozen fractions and the active ice nucleation site densities.

Aging Snomax^®^ samples at room temperature reduced the IN activity by a small amount which resulted in the reduction of the IN temperature curve when compared to the fresh samples. However, changing the duration of aging from 7 to 45 days did not show an appreciable difference, indicating that the reduction in IN activity had already taken place before the 7-day measurement had been made. Perhaps a much longer aging of the sample at room temperature is required before it can degrade enough to cause a large shift in the frozen fraction data. As a result, speeding up and amplifying the degradation was attempted using heat treatment. We also measured the secondary structure of the ice nucleating protein *inaZ* which is primarily responsible for templating water molecules to form ice and lowering the nucleation barrier [69,70]. It has been reported that the β-helix region of this protein is the primary IN site [71,72] and its presence can be detected using FTIR techniques [73] from the amide—I region of its IR spectra. A study into the amide—I region of purified IN proteins from Snomax^®^ was published recently [74], where the authors concluded that heating above 55 °C caused irreversible changes to the protein structure which resulted in reduced IN activity. We demonstrated for the first time that heat treatment causes most of the β-helix secondary structure in untreated Snomax^®^ to convert to a β-sheet or strand like structure using FTIR, and our microfluidic setup was able to show an associated decrease in the IN temperature. Furthermore, we showed that the extent of heat treatment correlated with the β-helix conversion and IN temperature reduction. Aged Snomax^®^ samples were not studied in the FTIR-ATR. While the effects of heat treatment on proteins are straightforward, aging in solution requires the proteins to interact with the solvent in a more direct way than heat treatment. Additionally, literature suggests that D_2_O has a different effect on the stability of certain proteins than H_2_O [75]. As a result, studying aged Snomax^®^ would require creating a dilute solution in H_2_O and studying the sample in transmission mode. This would be an interesting measurement in future work with biological INPs.

## 5. Conclusions

In summary, we developed a flow-through, temperature-controlled microfluidic platform with automated algorithms for ice nucleation detection. The custom MATLAB applications for the detection algorithms enable new users without specific knowledge of the codes or the algorithms to use the device for making measurements. We performed a case study with Snomax^®^ INPs and compared the performance of the two automated algorithms presented here. We also performed an investigation into the molecular nature of Snomax^®^ IN activity with FTIR and showed the effect of heat on the secondary structure of the ice nucleating protein, and measured the corresponding IN activity reduction in our microfluidic platform. It was found that aging at room temperature and mild heating at 55 °C had a minor detrimental impact on the ice nucleation ability of the Snomax^®^. However, heating at 95 °C reduced the ice nucleating ability of Snomax^®^ drastically. The DNN app enabled us to classify millions of droplets without human intervention and generated datasets that show congruence with literature data on Snomax^®^. Future studies will include measurements of heterogeneous ice nucleation activity of less efficient INPs as well as homogeneous ice nucleation temperature of pure water, which could be made possible with modifications in the carrier fluid and pumping setup. Ultimately, our device with automated detection could be modified to allow for active sorting of droplets containing INPs with the help of actuation pumps in future for aiding chemical and biological analysis of INPs downstream of our device.

## Data Availability

The data sets for this paper are publicly available in the Center for Aerosol Impacts on Chemistry of the Environment (CAICE), UC San Diego Library Digital Collections. Available online: https://doi.org/10.6075/J02N50T8 (Date accessed on 8 February 2021) [76].

## Supplementary Materials

The following electronic supplementary information (ESI) is available online at https://www.mdpi.com/2072-666X/12/3/296/s1: (1) A document with detailed descriptions of fabrication and calibration of the platinum resistive temperature devices, estimate of the temperature distribution of the microfluidic flow channel and the droplets from numerical simulation (Figures S1 and S2), plot of complete FTIR spectra of untreated and heat treated Snomax^®^ (Figure S3), further details and instructions on how to use the MATLAB codes for the DNN and polarized detection schemes (Figures S4–S8). (2) The following videos (a) Droplet Generation Video.AVI: This video shows droplet generation at the flow focusing junction of the microfluidic device. The video was captured at a framerate of 6400 fps and played back at 30 fps. (b) Droplet Freezing Video.AVI: This video shows ice nucleation and progression of crystallization of a droplet in the cold region of the microfluidic channel. The video was captured at a framerate of 10,000 fps and played back at 30 fps. (c) ROI Cropped Video.AVI: This video shows a cropped region of interest (ROI) which is used for detecting frozen droplets using a machine learning algorithm. (d) Droplet tumbling in Brightfield.AVI: This video shows droplets crystallizing and reorienting in flow in bright field. (e) Droplet tumbling in Polarized.AVI: This video shows droplets crystallizing and reorienting in flow in polarized light.
