# Supplementary material for: A Microfluidic Device for Automated High Throughput Detection of Ice Nucleation of Snomax®
Source: Micromachines (Basel). 2021 Mar 11;12(3):296. doi: 10.3390/mi12030296 (PMC7998955; doi:10.3390/mi12030296)
Supplement: Supplementary file 1 [file micromachines-12-00296-s001.zip › Micromachines Supplementary_Proof.pdf]

Electronic Supplementary Information (ESI)

for

A Microfluidic Device for Automated High Throughput Detection of Ice Nucleation of Snomax®

Priyatanu Roy[1], Margaret L. House[2], Cari S. Dutcher[*1, 2]

[1] Department of Mechanical Engineering, University of Minnesota - Twin Cities, Minneapolis, MN 55455;

[2] Department of Chemical Engineering & Materials Science, University of Minnesota - Twin Cities;

* Correspondence: cdutcher@umn.edu

Abstract

This electronic supplementary material contains: 1) Detailed descriptions of fabrication and calibration of the platinum resistive temperature devices (PRTD); 2) Estimate of the temperature distribution of the microfluidic flow channel and the droplets from numerical simulation; 3) Plot of complete FTIR spectra of untreated and heat treated Snomax; 4) Details of the MATLAB codes for the polarized and the deep neural network based freezing detection algorithms.

The following videos are also provided:

Droplet Generation Video.AVI: Shows droplet generation at the flow focusing junction

Droplet Freezing Video.AVI: Shows frozen droplets flowing through the micro channel

Droplet tumbling in Brightfield.AVI: Shows droplets crystallizing and reorienting after nucleation in bright field illumination

Droplet tumbling in Polarized.AVI: Shows droplets crystallizing and reorienting after nucleation in polarized illumination

1. Fabrication and calibration process of the PRTD arrays

The PRTD arrays were fabricated on 4” diameter, 0.5mm thick soda-lime glass wafers (University Wafers). First, they were cleaned with a Piranha solution (5:1 ratio of H_2SO_4 : H_2O_2) at 120°C for 15 minutes. Following this, they were washed with DI water in a dump rinser for three cycles and dried in a spin rinse dryer (SRD) for three 5 minute cycles. Then, the wafers were placed on top of a 200°C hot plate for 15 minutes for a dehydration bake. To start the spin coating, the wafers were primed with HMDS vapor for 3 minutes to promote adhesion of the photoresist. AZ1518 (Microchemicals GmBH), a positive photoresist was applied on a spin coater with 3300 rpm for 30 seconds at 3000 rpm/s ramp to get 2μm coating thickness. Pre-baking was done on a hotplate at 100°C for 50 seconds. Following this, a chrome photomask of the PRTD array was loaded in a MA6 mask aligner (Karl Suss). The wafer was exposed in hard contact mode for 9 seconds. Development was done by immersing the exposed wafer in Microposit 351 (Shipley). The developed wafer was rinsed with DI water and blow dried with N_2. Post bake was done for 50 seconds on a 115°C hotplate.

The PRTD arrays were sputter coated in an AJA sputterer by using DC powered metal targets. First, a 40 second step with a Ti target was used to coat the exposed wafer with a 2 nm layer to promote adhesion of the overlaying Pt layer. Following this, a 480 second step with a Pt target was used to achieve a layer depth of 200nm. Lift off lithography to remove the photoresist was performed overnight by immersing the wafer upside down inside a wafer carrier inside a vessel filled with acetone and placed on a hotplate at 90°C. Following this, excess flakes of photoresist and metal were removed with an airbrush gun filled with acetone. The thin-film thickness was measured with a P16 surface profiler (KLA Tencor). The wafer was annealed in a Mini-Brute tube furnace overnight at 500°C with N_2 purge flow to lower the film resistance and stabilize for use over longer durations. Following this, the wafer was cut using a wafer saw (DISCO) into 51x27mm individual rectangles with one PRTD array each. Finally, the leads of the PRTD array were masked with Kapton tape and a PECVD was used to coat the sensing region with a 200nm layer of SiO_2. This step ensured proper bonding of the PDMS to the substrate since PDMS cannot readily bond to the metal thin film.

The PRTD leads were connected to a custom PCB (Sunstone circuits) by using electrically conductive adhesive transfer tape (9703, 3M). A ribbon cable was used to connect the PCB to a Keithley 2701 digital multimeter with a 7710 multiplexer card. The resistance was measured by a four-wire method by sending a known current through the array and measuring the voltage drops across each pair of PRTD terminals.

The PRTD arrays were calibrated using a factory calibrated standard PRTD (Fluke 5606-50-B, accuracy ±0.04°C). A small aluminum vessel with base dimensions of 5x6cm was created from 1/8” sheet aluminum and placed directly on the temperature-controlled plate of the LTS 420 stage (Linkam). The PRTD array with the electrical connectors was placed, inside with the precalibrated standard PRTD placed in close proximity. The vessel was filled with methanol to ensure good heat transfer and

temperature uniformity between the array and the standard. The top was sealed with parafilm to prevent significant methanol evaporation. The LTS 420 stage was programmed with a temperature profile from -50 to +20°C with +10°C steps. Each temperature was held for 3 hours for the temperature distribution between the PRTD array and the standard to reach a uniform and steady value. Individual parabolic curves were fitted to the resistance of each PRTD in the array and the overall temperature accuracy from the fitted curves was within ±0.03°C.

2. Temperature distribution inside the microfluidic channel

During a typical experiment, the temperatures of five cold zones were adjusted until an isothermal profile was reached. Since the freezing detection region of interest was at the end of the cold region, small variations in the isothermal channel could lead to errors in reporting the exact temperature of freezing. While multiple experimental videos for a given sample recorded a fixed temperature data point, due to small fluctuations in the flow channel in the isothermal region during the recording, the overall uncertainty in the droplet temperature was estimated as ±0.26°C.

We also wanted to check if there was any significant temperature difference between the bottom, middle and the top of the channel. To achieve this without using a second set of sensors at the top of the channel, the temperature distribution of the microfluidic channel was modeled in COMSOL Multiphysics.

The model consisted of two distinct steps. The first step was to calculate the channel temperature and check for large variations across the channel at a given location. This was done by using a steady state laminar viscous flow model in COMSOL assuming a continuous flow of carrier fluid. The input to the model was the measured PRTD array temperature on the floor of the channel and the flow inlet temperature of 5°C which was maintained by the inlet cooling block.

The second step was to use the calculated channel fluid temperatures from the first step and apply them as a transient temperature boundary condition on the droplet surface. The rationale behind this was that from the reference frame of the moving droplet, the channel temperature would be experienced as a function of time. For this step, the channel and the continuous fluid were not modeled. The reported droplet temperature was the average temperature of the whole droplet.

Figure S1 shows that there is a difference of less than 0.1°C between the bottom and the centerline of the channel, where the droplets flow through, at an isothermal channel temperature of about -5°C. This nonuniformity grew to about 0.2°C at -20°C but was still smaller than the experimental temperature uncertainty limit of 0.26°C as mentioned previously. As a result, we directly used the temperature measured by the PRTDs as the channel temperature for the droplet temperature calculation in step 2.

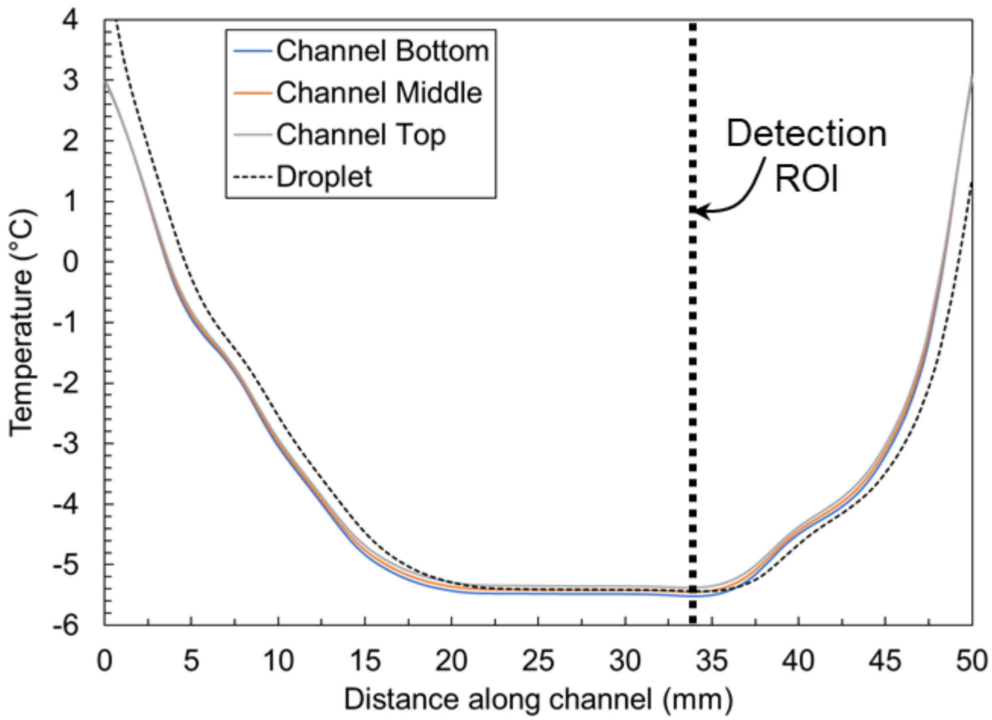

Figure S1: Temperature distribution in the microfluidic flow channel and droplet. Droplet diameter 70 µm, droplet and carrier fluid flow velocity 15 mm/s. The droplet resides inside the isothermal zone for ~1 second.

Also shown in Figure S1 is the droplet temperature as it moves through the channel calculated from step 2. There is a temperature lag in the droplet compared to the channel temperature in the region where the temperature drops sharply before the droplet reaches the isothermal region maintained by the individually controlled cold zones. The approximately 1 second of residence time in the isothermal region enables the droplet to equilibrate with the channel temperature and lets us directly report this temperature as the droplet temperature for the frozen fraction curves. The temperature of the droplet was also simulated at additional channel temperatures of -10, -15 and -20°C. The results are shown in Figure S2. There was no appreciable difference between the droplet temperature and the recorded PRTD temperatures.

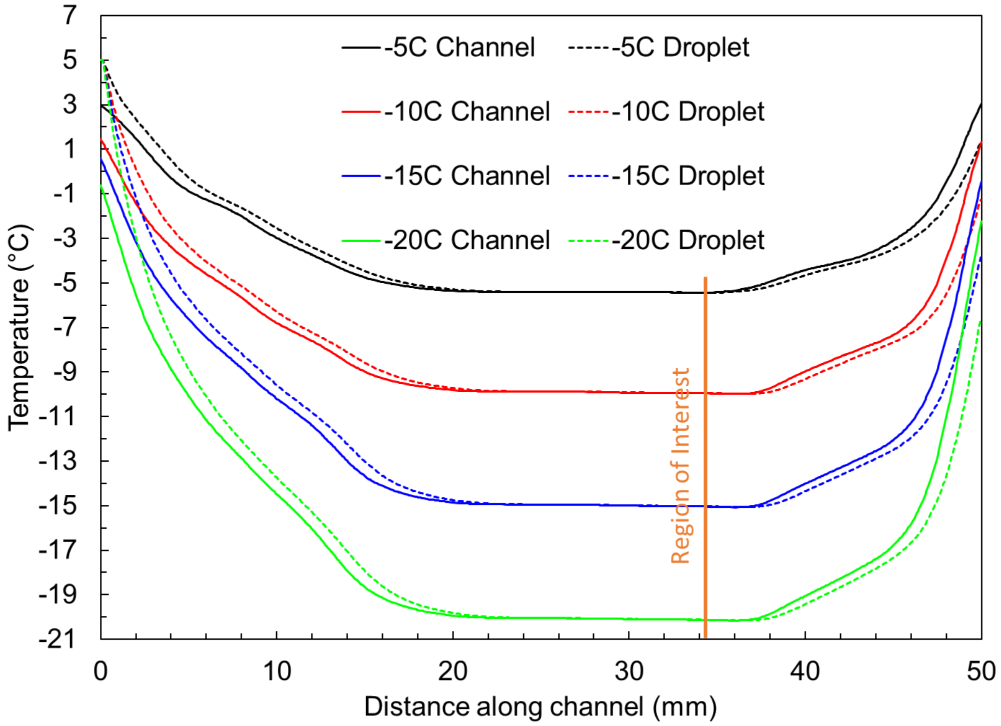

Figure S2: Droplet temperatures inside flow channel at different isothermal channel temperatures. The orange line indicates the region of interest where frozen droplets were counted

3. FTIR Spectra of untreated and heat treated Snomax

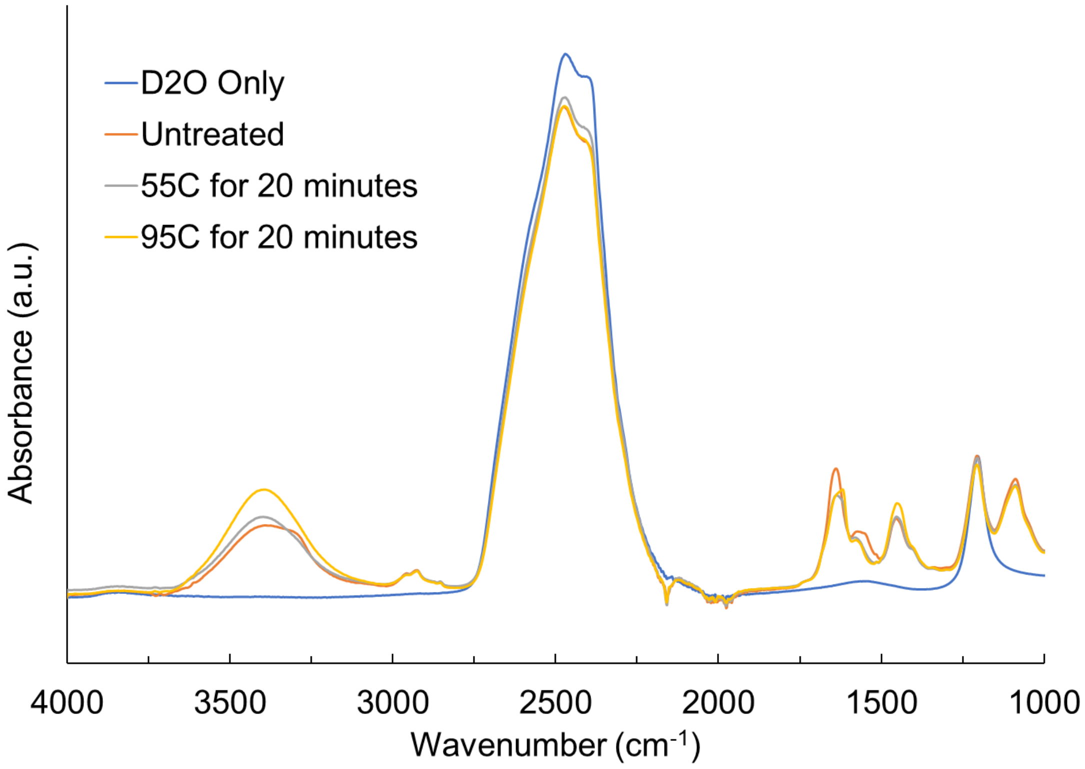

Figure S3: Complete FTIR spectra of untreated and heat treated Snomax in D_2O

Figure S3 shows the absorbance spectra of the heat treated Snomax samples in D_2O and a spectrum without any Snomax. The difference between the samples is apparent in the absence of peaks near the 3000-4000 cm^{-1} and 1000-2000 cm^{-1} regions in the D_2O samples without Snomax. The largest peak in all the samples is the O-D stretch of the D_2O at 2200-2700 cm^{-1}. In samples with Snomax the strong O-H band is visible in the 3200-3600 cm^{-1} region. The differences between the curves at the 1500-1700 cm^{-1} is ascribed to the proteins present in the Snomax samples. This region is further subdivided into Amide-I (1600-1700 cm^{-1}) and Amide-II (1500-1600 cm^{-1}) regions. The Amide-I peaks are due to the C=O bond vibrations and the Amide-II peaks are due to C-N stretching. The C=O bonds in a protein are attributed to the secondary structure of the protein, and as a result this regions was chosen in our study to represent the effect of heat on the secondary structure and corresponding decrease in ice nucleating ability.

4. MATLAB Codes for DNN and polarized droplet freezing detection algorithm

DNN detection algorithm

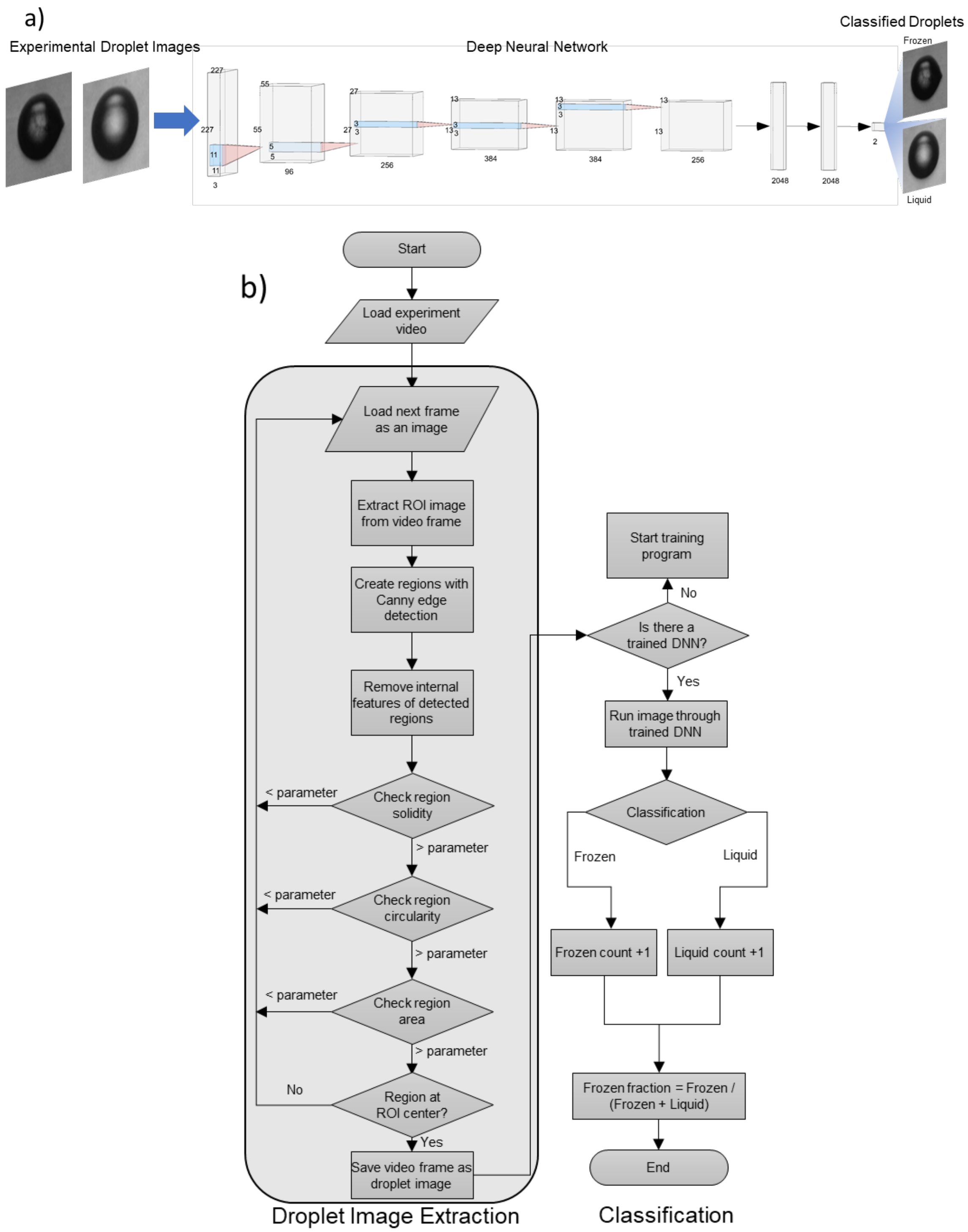

Figure S4: (a) Workflow for polarized threshold selection for automated classification (b) Flowchart of threshold selection using a parametrization approach and droplet classification.

Figure S4a, b shows the DNN layers and the flowchart for the detection algorithm. An experimental video is loaded into the app. The app reads each frame of the video, performs segmentation to find the droplet inside the ROI using a quick Canny edge detection followed by checking region solidity, circularity and area to cancel out lighting artifacts from the video frames. When a droplet is found, it is tracked until the droplet reaches the center of the ROI and the image is extracted for classification. Depending on the options selected in the app, it can also save the image in a folder without classification. Extracting images from the full video without classification is useful for creating the training dataset and reducing the storage space required for the experimental data. The training dataset consists of around 10,000 to 15,000 images of liquid and frozen droplets for each experiment. These images are split randomly into 60% training, 20% validation and 20% test data. Minibatch stochastic gradient descent is performed with batch size of 128, validation is performed every 40 iterations and the maximum number of epochs is set to 10.

DNN code usage instructions

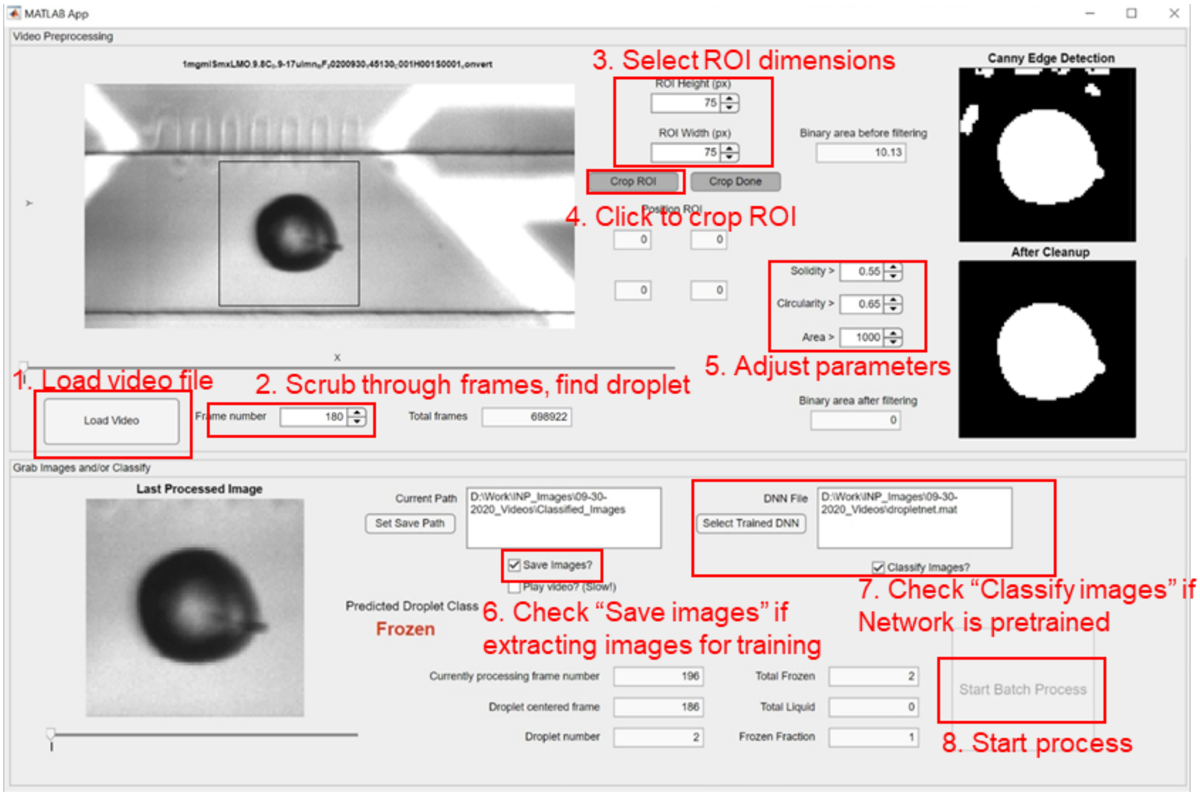

Figure S5: MATLAB GUI app to crop video, extract droplet images and classify them using a DNN

The main app INP_Image_Cropper_Grabber_Classifier.mlapp processes videos, extracts images, saves images, and if provided with a pretrained network, also classifies and gives a running frozen fraction count. To use this app, the user first loads videos taken with the highspeed camera and converted to AVI format. Then the user scrubs through the video frames either by using the slider or by entering a frame number in the indicated box until a droplet is fully in view. Then the user enters an ROI size such that it is larger than

a single droplet. Then the user clicks the crop ROI button, moves the ROI window with the mouse and clicks on a droplet. Following this, the binarized droplet image is shown in the frame titled 'Canny Edge Detection'. At this stage there may be some other artifacts left in the frame. The user adjusts the solidity, circularity and area parameters until only the droplet is visible in the 'After Cleanup' frame. The user repeats this for a few droplets to make sure the cleaned up image only shows the droplet and nothing else. When satisfied with the results, the user selects a save path to save extracted images, if desired. Note that this step is essential when extracting images to train a network. If a pretrained network exists, the user selects the trained DNN and checks the corresponding checkbox. Finally, the user clicks the "Start Batch Process" button. The code runs through all the video frames, crops to the ROI, selects images where the droplet is centered in the ROI, saves it, runs it through a trained DNN (depending on options selected). A running count can be seen in the textboxes to the left of the Start Batch Process button.

Manual image classification

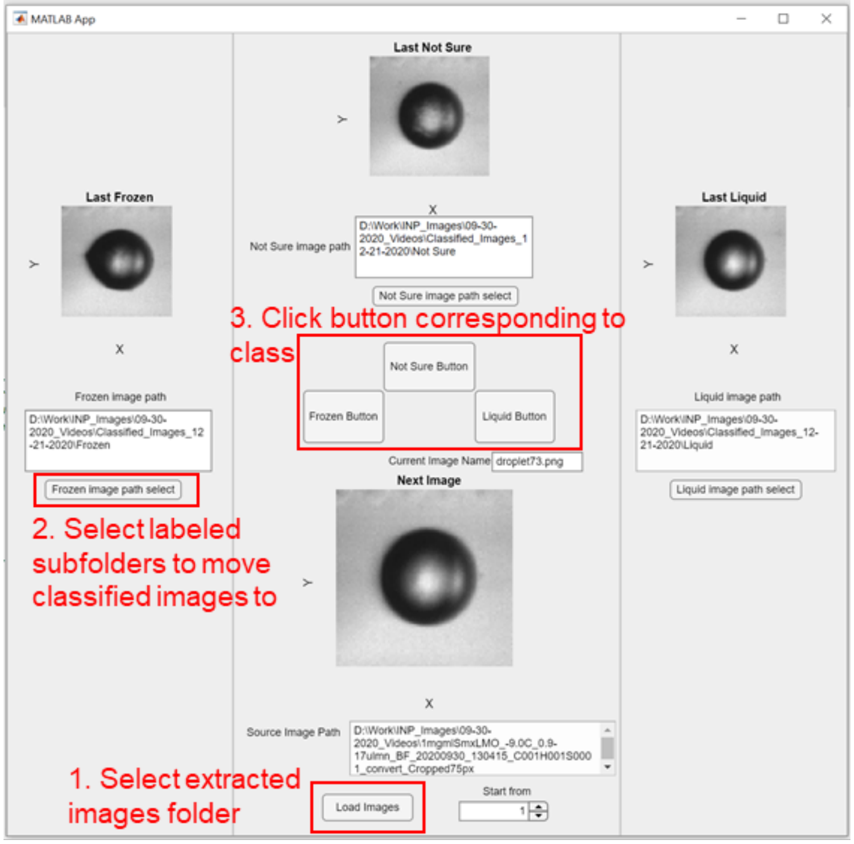

Figure S6: MATLAB GUI app to manually classify images

This is a secondary app *INP_Image_Sorter.mlapp* which is useful for classifying droplet images saved through the primary app. The user first selects the folder where droplet images are saved. Then the user selects the paths for "Liquid", "Frozen" and "Not Sure" (i.e. droplets which cannot be placed in the liquid or frozen class at a first glance) folders. The first image in the source folder should be loaded in the Next

Image frame. If not, the user can select the image number from the “Start from” list. Once the image is visible, the user can click the button in the middle corresponding to a class. The next image should load and the process can be repeated to move through the images in the source folders. Note that the process can be resumed from any numbered droplet image in the source folder by changing the “Start from” value.

The code to train the network *dropletnetcode.m* has been added as part of the ESI. Images classified with these apps can be used to train the network and used for classification.

Polarized detection algorithm

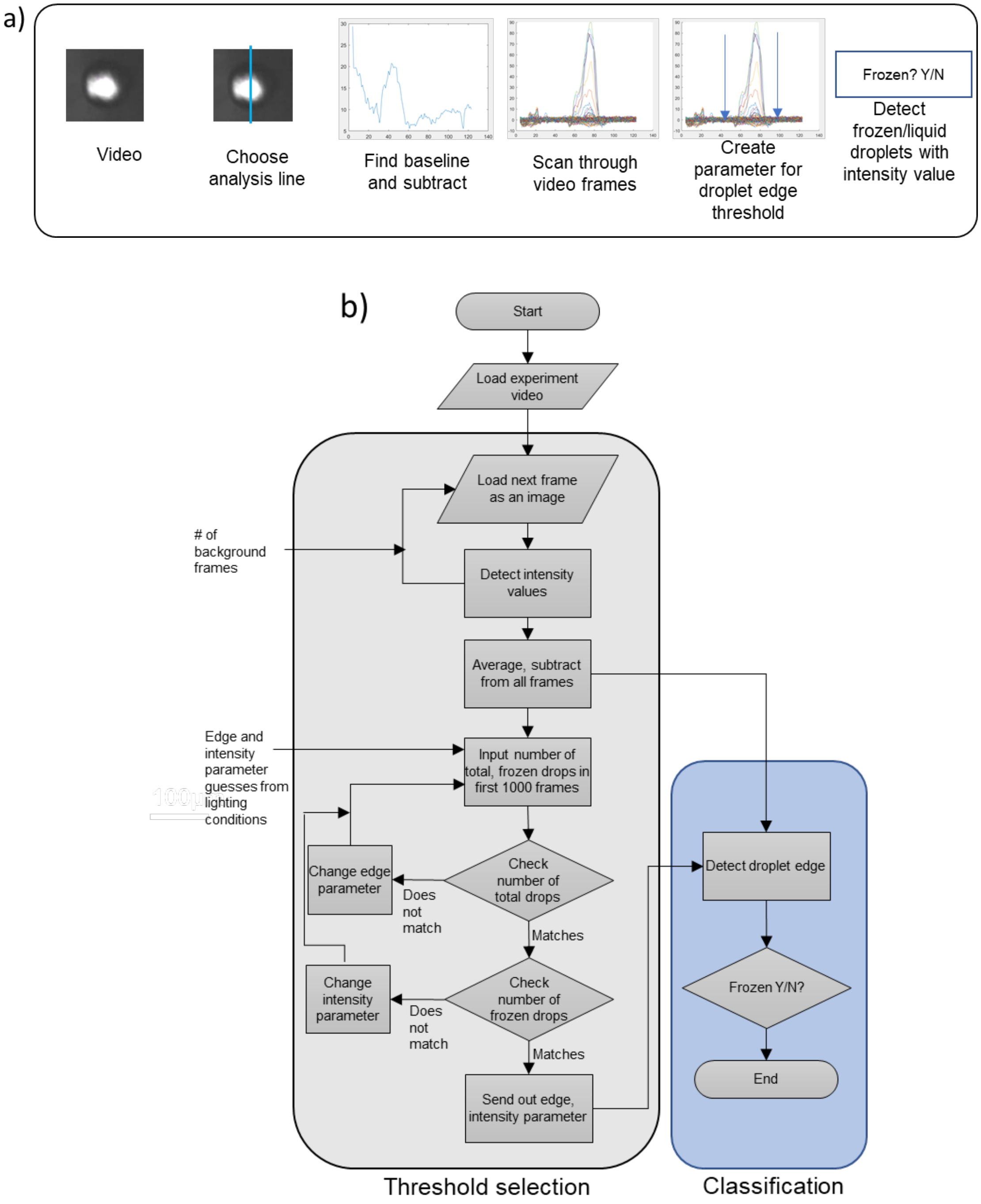

Figure S7: (a) Workflow for polarized threshold selection for automated classification (b) Flowchart of threshold selection using a parametrization approach and droplet classification.

Figure S7a, b shows the flowchart of the polarized detection code. Background light from the environment and the device is subtracted to give a baseline that only includes deviations due to the droplets passing through the line of analysis. In the initial parameterization section of the code, the user

inputs the number of frozen droplets and the number of total droplets that pass through the line of analysis within the first 1000 video frames. While this step could be optional, this manual input allows for the optimization of an "edge" parameter that determines the threshold for the beginning and end of the droplet, as well as an "intensity" parameter that determines the dual intensity thresholds the droplet must meet to be considered frozen. The intensity parameter acts like a band pass filter, with an upper and a lower threshold. Liquid droplets fall between the two thresholds while frozen drops are either darker or brighter than the threshold region. The edge and intensity parameters are changed until the number of droplets counted by the code matches the user input for this short portion of the video (typically 30 seconds when played back at 60 fps, and around 10 droplets).

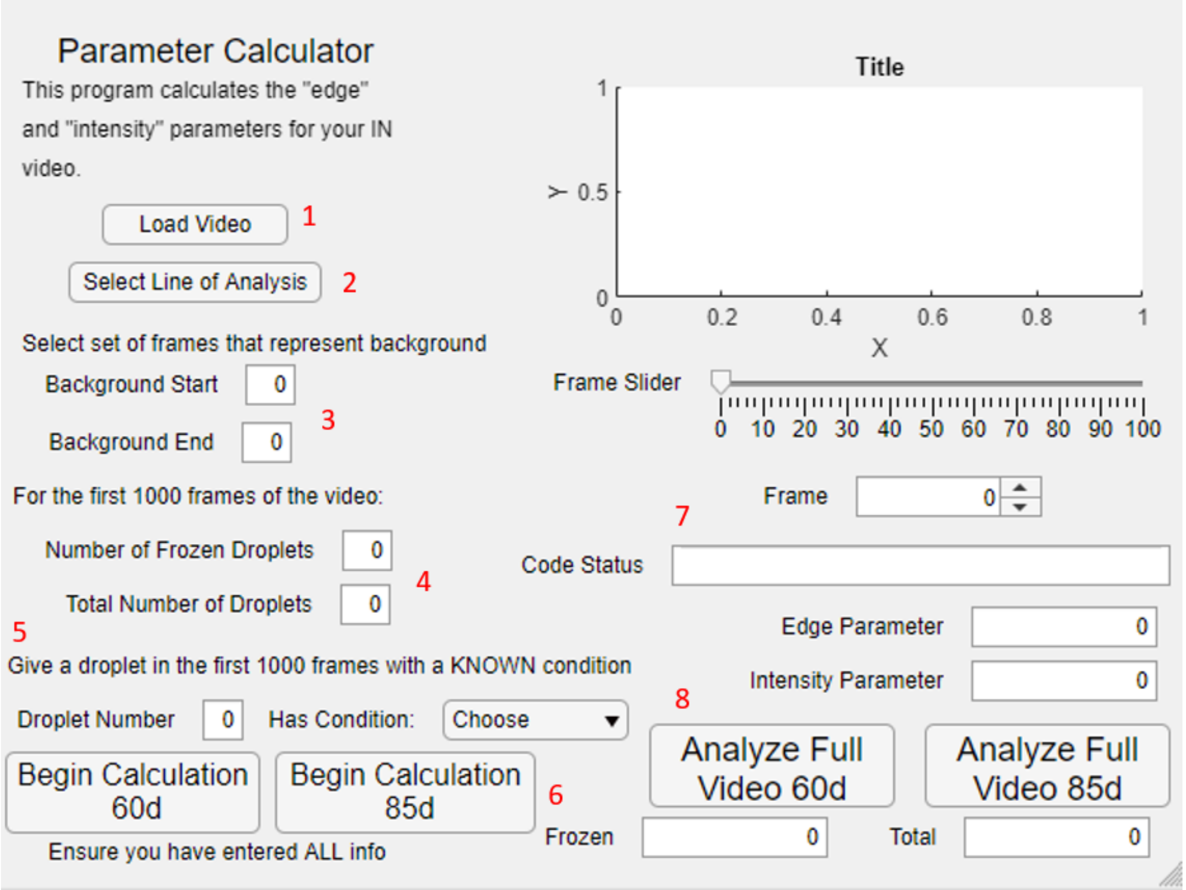

Figure S8 MATLAB GUI app for polarized detection

Polarized code usage instructions

The MATLAB app for the polarized freezing detection code is called "parameter_calculator.mlapp".

1. Click the "Load Video" button and select your video. Be sure the file is in AVI format.
2. Click the "Select Line of Analysis" button. A figure will pop up showing the first frame of your video. Click once where you would like your Y axis line of analysis to begin and click again were you would

like it to end. The tool will not stop drawing a line after this. Press enter to confirm your line selection and exit the figure.

3. Under “Select set of frames that represent background”, choose a range of at least 20 consecutive frames in which the only the background is in the line of analysis. Type in the number of the first frame under “Background Start” and the number of the last frame under “Background End”.
4. Under “For the first 1000 frames of the video”, type in the number of frozen droplets that pass through the line of analysis completely in the first 1000 frames of the video and the total number of droplets that pass through the line of analysis completely in the first 1000 frames of the video.
5. Under “Give a droplet in the first 1000 frames with a KNOWN condition”, choose a droplet in the first 1000 frames of the video that you know to be either a liquid droplet or a bright frozen droplet. Type in the number of this droplet as it appears in the video and select its state from the drop-down menu. For example, if the third droplet in the video is liquid, type “3” into “Droplet Number” and choose “Liquid” from the drop-down menu.
6. For a video taken at or close to 60° polarizer angle, click “Begin Calculation 60d”. For a video taken at or close to 85° polarizer angle, click “Begin Calculation 85d”.
7. Observe the “Code Status” bar for updates. You will see “Edge Parameter” change first, and then “Intensity Parameter”, before the code finishes. If this step takes longer than 10min, try repeating steps 1 – 6 with a slightly different line of analysis.
8. Once the “Code Status” bar reads “Finished”, click “Analyze Full Video 60d” for a 60° video or click “Analyze Full Video 85d” for an 85° video. The number of total droplets will populate first in “Total”, followed by the number of frozen droplets in “Frozen”. The code is finished when neither number changes for a period of 1min.
